# Evaluating the International Classification of Functioning, Health, and Disability Model as an Aging Model

**DOI:** 10.1093/geroni/igaa057.1643

**Published:** 2020-12-16

**Authors:** Qiwei Li, Becky Knight

**Affiliations:** University of North Texas, Denton, Texas, United States

## Abstract

The study provides a possible theoretical framework for future aging studies focusing on a comprehensive understanding of the relationship between physical functions, social participation, and context factors including environmental and personal variables. The International Classification of Functioning, Health, and Disability (ICF) model has received considerable studies in rehabilitation counseling fields as it bridges the gap between functional limitations and overall health status for social participation. The ICF model focuses beyond physical conditions and embraces social supports and personal coping styles. This study verifies the validity of the ICF model with a data set collected from a fall prevention program. For the methods, a structural equation modeling was estimated with latent variables including body structure, body functions, activities, and personal factors. The latent variables were suggested by the ICF framework. The results showed that the estimation outcome exhibited an acceptable goodness of fit, χ2(11) = 30.401, p = .001 (due to the large sample size of 691), RMSEA= .051 [.030, .072], CFI = .968, TLI = .919, SRMR = .029. The equation level good of fit also was great with an overall R squared of .828. In conclusion, the ICF model was valid and has been tested in the aging studies with the data set collected from a fall prevention program for older adults. As the ICF model includes more variables than medical models such as personal attributions, a holistic understanding regarding aging experience among older adults from various backgrounds will become possible, which is an urgent need for diverse America.

